# Malignancies in the rheumatoid arthritis abatacept clinical development programme: an epidemiological assessment

**DOI:** 10.1136/ard.2008.097527

**Published:** 2008-12-01

**Authors:** T A Simon, A L Smitten, J Franklin, J Askling, D Lacaille, F Wolfe, M C Hochberg, K Qi, S Suissa

**Affiliations:** 1 Global Epidemiology, Bristol-Myers Squibb, Hopewell, New Jersey, USA; 2 ARC Epidemiology Unit, University of Manchester, Manchester, UK; 3 Epidemiology, Karolinska University Hospital Solna, Stockholm, Sweden; 4 Arthritis Research Centre of Canada, University of British Columbia, Vancouver, Canada; 5 National Data Bank for Rheumatic Diseases, Arthritis Research Foundation and University of Kansas, Wichita, Kansas, USA; 6 Departments of Medicine and Epidemiology and Preventive Medicine, University of Maryland School of Medicine, Baltimore, Maryland, USA; 7 Global Biostatistics, Bristol-Myers Squibb, Hopewell, New Jersey, USA; 8 Division of Clinical Epidemiology, McGill University, Montreal, Canada

## Abstract

**Objective:**

To provide context for the malignancy experience in the rheumatoid arthritis (RA) abatacept clinical development programme (CDP) by performing comparisons with similar RA patients and the general population.

**Methods:**

Malignancy outcomes included total malignancy (excluding non-melanoma skin cancer (NMSC)), breast, colorectal, lung cancers and lymphoma. Comparisons were made between the observed incidence in patients within the abatacept CDP and RA patients on disease-modifying antirheumatic drugs (DMARD) identified from five data sources: the population-based British Columbia RA Cohort, the Norfolk Arthritis Register, the National Data Bank for Rheumatic Diseases, the Sweden Early RA Register and the General Practice Research Database. Age and sex-adjusted incidence rates (IR) and standardised incidence ratios (SIR) were used to compare events in the abatacept trials with the RA DMARD cohorts and the general population.

**Results:**

A total of 4134 RA patients treated with abatacept in seven trials and 41 529 DMARD-treated RA patients in the five observational cohorts was identified for study inclusion. In the abatacept-treated patients, the 51 malignancies (excluding NMSC), seven cases of breast, two cases of colorectal, 13 cases of lung cancer and five cases of lymphoma observed were not greater than the range of expected cases from the five RA cohorts. The SIR comparing RA patients with the general population were consistent with those reported in the literature.

**Conclusions:**

The IR of total malignancy (excluding NMSC), breast, colorectal, lung cancers and lymphoma in the abatacept CDP were consistent with those in a comparable RA population. These data suggest no new safety signals with respect to malignancies, which will continue to be monitored.

Abatacept is the first in a class of agents for the treatment of rheumatoid arthritis (RA) that selectively modulates the CD80/CD86 : CD28 co-stimulatory signal required for T-cell activation.^1^ Abatacept has demonstrated efficacy in the treatment of RA.^2^ ^3^ ^4^ ^5^ Although abatacept has also demonstrated a favourable safety and tolerability profile in RA clinical trials, its potential risk for rare adverse events such as malignancies has not been addressed in the published literature. When a new medication becomes available, there is always some level of concern for the long-term safety of the medication in a broader patient population.

The risk of malignancy events is of particular importance in patients who receive immunomodulatory therapies, such as biological disease-modifying antirheumatic drugs (DMARD).^6^ The current analysis was part of a premarketing risk assessment that focused on malignancies occurring in patients in the abatacept clinical development programme (CDP).^7^ To place these observations into context, we compared the data from abatacept-treated patients with data on malignancies from existing RA cohorts and the general population. A recent meta-analysis suggested that RA patients may be at higher risk of some site-specific malignancies than the general population, in particular lymphoma and lung cancer, thus making RA patients a more appropriate comparison group than the general population.^8^

## Methods

### Study design

This observational study examining malignancies was based on the comparison of cancer occurrence in patients exposed to abatacept within the CDP, cancer occurrence in five observational cohorts of RA patients in Europe and North America and the occurrence of malignancies in the general population.

### Data sources

Clinical safety data from seven abatacept RA clinical trials were included in the analyses. Table 1 presents these studies.^3^ ^9^ ^10^ ^11^ ^12^ ^13^ ^14^

**Table 1:** Description of the abatacept clinical trials included in the current analysis

Study name	Study design	Malignancy screening and exclusions	Duration of double-blind period (months)	Abatacept	Placebo	Open-label extension
	Study title					
IM101100 Phase IIB	Randomised, dose-ranging, placebo-controlled, double-blind	Mammography requiring further investigation. Complete evaluation before dosing; history of cancer within the past 5 years; excluding NMSC cured by local resection	12	220	119	219
IM101101 Phase IIB	Randomised, placebo-controlled, double blind	Mammography requiring further investigation. Complete evaluation before dosing; history of cancer within the past 5 years; excluding NMSC cured by local resection	12	85	36	80
AIM IM101102 Phase III	Randomised, placebo controlled, double-blind; Abatacept in inadequate responders to methotrexate	Subjects with a mammogram that is suspicious for malignancy; history of cancer within the past 5 years; excluding NMSC cured by local resection	12	433	219	539
ASSURE IM101031 Phase III	Randomised, placebo-controlled, double-blind; Abatacept Study of Safety in Use with other RA thErapies	Subjects with a mammogram that is suspicious for malignancy; history of cancer within the past 5 years; excluding NMSC cured by local resection	12	959	482	1184
ATTAIN IM101029 Phase III	Randomised, placebo-controlled, double-blind; Abatacept Trial in Treatment of Anti-TNF INadequate responders	Subjects with a mammogram that is suspicious for malignancy; history of cancer within the past 5 years; excluding NMSC cured by local resection	6	258	133	317
	Total double-blind 5 core above			1955	989	2689†
ATTEST IM101043	Abatacept or infliximab versus placebo, a Trial for Tolerability, Efficacy and Safety in Treating RA	History of cancer within the past 5 years; excluding NMSC cured by local resection	12	156	110	236* (132 abatacept, 104 placebo, 136 infliximab)
ARRIVE IM101064	Abatacept Researched in Rheumatoid arthritis patients with an Inadequate anti-TNF response to Validate Effectiveness	History of cancer within the past 5 years; excluding NMSC cured by local resection	6 (open-label)	1046		530

*IM101043, without infliximab arm; †Number represents total number of abatacept exposed patients exposed during both double-blind and open-label; five core trials N  =  2689; overall N  =  4134.

NMSC, non-melanoma skin cancer; RA, rheumatoid arthritis; TNF, tumour necrosis factor.

For the RA comparison groups, analyses were performed on data from five RA cohorts. These cohorts were derived from the population-based British Columbia (BC) RA Cohort in Canada, the Norfolk Arthritis Register (NOAR) in the UK, the National Data Bank for Rheumatic Diseases (NDB) in the USA, the Early Rheumatoid Arthritis Register in Sweden (Sweden ERA) and the General Practice Research Database (GPRD) in the UK. Characteristics of these data sources have been described previously in the literature.^15^ ^16^ ^17^ ^18^ ^19^ The five cohorts were selected for their ability to provide the patient population of interest (patients receiving non-biological DMARD only), to provide age and sex-specific incidence rates (IR) of the specified outcomes and their ability to complete the analyses for regulatory filings. Table 2 presents the characteristics of these databases.

**Table 2:** Characteristics of data sources used for identification of RA patients included in the epidemiological analysis

	BC	NDB	GPRD	NOAR	Sweden ERA
Country	Canada	USA	UK	UK	Sweden
Data type	Administrative data on physician visits, hospitalisations and medications	Patient questionnaire	Electronic medical records	Patient questionnaire and assessment	Electronic medical records, patient assessment
Time period	1996–2001	1998–2003	1987–2001	1990–9	1994–2003
No of RA patients in cohort	27 710	21 229	38 622	839	3703
Type of cohort	Population-based, prevalent cases	Longitudinal cohort, prevalent cases referred by rheumatologists	Population-based, prevalent cases	Early RA cohort	Early RA cohort
No of DMARD-treated RA patients	12 337	10 499	14 467	523	3703
DMARD users	Prevalent users	Prevalent users	Prevalent users	Incident users	Incident users
Case ascertainment	ICD-9 codes on claims and discharge summaries	Patient-reported and verified by medical and hospital records	OXMIS codes; codes validated by an oncologist	ICD-9 codes in linked medical records	ICD-10 codes and verified by linking to cancer registry

BC, British Columbia RA Cohort; DMARD, disease-modifying antirheumatic drug; GPRD, General Practice Research Database; ICD, International Classification of Diseases; NDB, National Data Bank for Rheumatic Diseases; NOAR, Norfolk Arthritis Register; RA, rheumatoid arthritis; Sweden ERA, Sweden Early Rheumatoid Arthritis Register.

For the general population comparison group, data on malignancies were obtained from the Surveillance Epidemiology and End Results (SEER) database, which provides age and sex-specific IR of malignancies for the US general population.^20^ We also reference a recent meta-analysis paper that evaluated a total of 17 publications examining standardised incidence ratios (SIR) of malignancy in RA patients compared with the general population or non-RA patients who met our inclusion criteria:^8^ five studies reported SIR for total malignancy excluding NMSC;^21^ ^22^ ^23^ ^24^ ^25^ nine for breast cancer;^21^ ^24^ ^25^ ^26^ ^27^ ^28^ ^29^ ^30^ ^31^ 10 for colorectal cancer;^21^ ^24^ ^25^ ^26^ ^27^ ^28^ ^29^ ^30^ ^31^ ^32^ 12 for lung cancer^21^ ^24^ ^25^ ^26^ ^27^ ^28^ ^29^ ^30^ ^31^ ^33^ ^34^ and six for lymphoma.^15^ ^28^ ^35^ ^36^ ^37^ ^38^

### Study subjects

Patients in the abatacept CDP included those who were randomly assigned to abatacept treatment during the double-blind period, as well as all patients receiving abatacept during open-label extension periods. The cumulative abatacept experience included 4134 abatacept-treated patients representing 8388 person-years of exposure in the seven clinical trials. A total of 1955 patients originated in the abatacept arms of the double-blind periods of the five core RA studies. Individuals who agreed to enter the open-label period after completing the double-blind period were enrolled; no specific response criteria or additional screening was required.

Because 80% of subjects in the abatacept CDP were on background DMARD therapy during the trials (usually methotrexate) and almost all had previous exposure to DMARD, the most relevant reference group for comparison with the abatacept patients was considered to be DMARD-treated patients. Consequently, non-biological DMARD cohorts were identified from each of the data sources. For the Sweden ERA cohort, the investigators estimated that 95% of the subjects received DMARD; therefore, this whole group was included as a DMARD cohort. In total, 41 529 patients with RA were included and the demographic characteristics of these patients are presented by cohort in table 3.

**Table 3:** Baseline demographics and clinical characteristics of abatacept clinical trial patients and RA DMARD cohorts included in the epidemiological analyses

	Abatacept Clinical Trial Program (N = 4134)	BC (N = 12 337)	NDB (N = 10 499)	GPRD (N = 14 467)	NOAR (N = 523)	Sweden ERA (N = 3703)
Age, years, n (%)						
<20	4 (0.1)	214 (2)	13 (0.1)	34 (0.2)	3 (1)	29 (1)
20–44	1011 (24)	2874 (23)	1429 (14)	2856 (20)	106 (20)	753 (20)
45–64	2435 (59)	5148 (42)	4870 (47)	6998 (48)	246 (47)	1624 (44)
65–74	553 (13)	2692 (22)	2716 (26)	3375 (23)	120 (23)	797 (22)
⩾75	131 (3)	1409 (11)	1438 (14)	1204 (8)	48 (9)	500 (14)
Female, n (%)	3323 (80)	8936 (72)	7971 (76)	10 284 (71)	357 (68)	2589 (70)
Duration of RA, years, n (%)						
<5	1353 (33)	4890 (40)	2726 (29)^*^	NA	523	3703
5–10	1192 (29)	4206 (34)	1902 (20)^*^	NA	0	0
>10	1586 (38)	3241 (26)	4716 (50)^*^	NA	0	0
Concomitant medications, n (%)^†^						
Oral corticosteroids	2657 (64)	8121 (66)	NA	6902 (48)	194 (37)	NA
NSAID	3113 (75)	11 001 (89)	6820 (65)	13 656 (94)	416 (80)	NA
Total follow-up, years						
Mean	2.1	4.9	3.3	3.7	7.9	3.6
Median	1.8	6.0	2.5	4.1	9.3	NA

^*^Rheumatoid arthritis (RA) duration was not collected for every subject in the NDB; therefore, n  =  9344 for this variable. ^†^Use of concomitant medications at baseline is presented for the abatacept trial population, whereas use during follow-up is presented for the RA cohorts (when available).

BC, British Columbia RA Cohort; DMARD, disease-modifying antirheumatic drug; GPRD, General Practice Research Database; NDB, National Data Bank for Rheumatic Diseases; NOAR, Norfolk Arthritis Register; NSAID, non-steroidal anti-inflammatory drugs; Sweden ERA, Sweden Early Rheumatoid Arthritis Register.

Across all cohorts, most patients (63–72%) were between 45 and 74 years of age and the proportions above and below this range were low and similar to each other. Patient age at cohort entry (baseline) was used for the early RA cohort NOAR. For cohorts with longer follow-up (BC and NDB) and the ERA, patient age was the age assigned at the time the event occurred. The BC and NDB cohorts were characterised by a population that consisted primarily of patients with established RA of greater than 5 years duration, 60% and 70% of the subjects, respectively, whereas the early RA cohorts followed patients from disease onset. Mean patient-years of follow-up across RA DMARD cohorts ranged from 3.3 to 7.9 patient-years.

### Case ascertainment

Prespecified outcomes included total malignancy excluding non-melanoma skin cancer (NMSC) and the four site-specific malignancies of breast cancer, colorectal cancer, lung cancer and lymphoma. Breast, lung and colorectal cancers were included because they represent the three most common cancer sites in women (in the USA). Lymphoma was included as it has consistently been shown to be elevated in RA patients.^9^ ^10^ ^11^ ^12^ ^13^ ^14^ ^15^ ^16^ ^17^ ^18^ As outlined in table 2, malignancies for this analysis were identified by International Classification of Diseases (ICD)-9 and ICD-10 diagnostic codes in the BC, NOAR and the Sweden ERA data sources, as reported within the registries or through linkage to cancer registers (Sweden ERA). OXMIS and Read codes recorded in the medical record were used to identify malignancies in the GPRD analyses. In the NDB, malignancies were identified from semi-annual questionnaires completed by RA patients who were subsequently validated by physicians. For patients in the abatacept CDP, malignancies were identified from all adverse event reports and validated through special event forms; events were included regardless of their relationship to the study drug.

### Analyses

Baseline demographics and clinical characteristics were computed using descriptive statistics for continuous or categorical variables as appropriate. In the abatacept clinical experience, exposure to abatacept and incidence of malignancy were counted from the start of therapy until the first event or end of treatment period plus 56 days, whichever occurred first. Rates were computed for the double-blind period, as well as the cumulative double-blind and open-label study period. In the RA DMARD observational cohorts, person-time and incidence of malignancy were calculated from the first recorded non-biological DMARD exposure until the first event or the end of follow-up, whichever occurred first. The IR for each outcome of interest in the RA DMARD cohorts were standardised to the age (10-year interval) and sex distribution of the abatacept clinical trial experience.

To estimate the relative risk of malignancy in the abatacept CDP relative to that in each of the five RA DMARD cohorts and the US general population, SIR were calculated, dividing the observed numbers of malignancy cases in the abatacept trial experience by the expected numbers. The expected numbers were calculated by multiplying the cancer rates in the five RA cohorts by the observed person-years at risk, stratified by sex and 10-year age group. Furthermore, we computed a summary SIR estimate (and 95% CI) combining the SIR from the five DMARD cohorts based on the meta-analysis method of DerSimonian and Laird.^39^ This method uses a random effects model that considers both within-study and between-study variation by incorporating the heterogeneity of effects in the overall analysis.

To determine if our results differ from those in the published literature, the SIR obtained were compared with those published in studies evaluating non-RA or general population malignancy incidence to RA populations.

For all SIR, 95% CI were calculated using the Wilson and Hilferty approximation. Statistical analyses were performed using the SAS software package.

## Results

The numbers and rates of malignancies in the abatacept CDP are shown in table 4.

**Table 4:** Incidence of malignancies in the abatacept clinical trial experience

	Placebo DB*†	Abatacept DB*†	Abatacept 5 core RA RCT† (DB+OL)‡	Abatacept cumulative§ (DB+OL)‡
No of patients	989	1955	2689	4134
No of person-years of follow-up	794	1688	7282	8388
Total malignancies (excluding NMSC)	5	10	46	51
	0.63 (0.26 to 1.5)	0.59 (0.32 to 1.1)	0.63 (0.46 to 0.84)	0.61 (0.45 to 0.80)
Breast cancer	2	1	5	7
	0.25 (0.06 to 1.01)	0.06 (0.01 to 0.43)	0.07 (0.02 to 0.16)	0.08 (0.03 to 0.17)
Colorectal cancer	0	0	2	2
			0.03 (0.00 to 0.10)	0.02 (0.00 to 0.09)
Lung cancer	0	4	13	13
		0.24 (0.09 to 0.64)	0.18 (0.10 to 0.31)	0.15 (0.08 to 0.27)
Lymphoma	0	1	4	5
		0.06 (0.01 to 0.43)	0.05 (0.01 to 0.14)	0.06 (0.02 to 0.14)

Unless otherwise indicated, values represent number of cancer cases and incidence rates per 100 person-years (95% CI). *DB, double blind; randomisation 2 : 1 abatacept to placebo. †Includes the five core rheumatoid arthritis (RA) studies: ATTAIN, ASSURE, AIM and two phase II studies. ‡OL, open label, December 2006 data lock. §Includes the five core RA studies and the ATTEST and ARRIVE studies.

NMSC, non-melanoma skin cancer; RCT, randomised controlled trial.

During the double-blind period, 10 malignancies were reported in the 1955 patients with 1688 person-years of follow-up receiving abatacept and five malignancies in the 989 patents with 794 person-years of follow-up randomly assigned to placebo. During the double-blind period, lung and breast cancers were the most frequently reported solid malignancies in the abatacept and placebo-treated patients, respectively.

Four cases of lung cancer were identified in the abatacept patients and none in the placebo group during the double-blind period. The cases of lung cancer presented with no atypical clinical features; all patients were over 60 years of age and three of the four subjects were heavy smokers. There was no predominant tumour type. Two of the cases were in subjects who had exposure to abatacept of 100 days or less (29 days and 100 days). A third subject had a small apical lung nodule that went undetected on the pretreatment chest radiograph.

There were two cases of breast cancer in the placebo group and one case in patients receiving abatacept. All three cases were identified at the annual protocol-required mammogram at one year. Two of the cases (one abatacept and one placebo) were noted to have normal mammography at baseline. One case in the placebo group did not report baseline mammography results. No cases of colorectal cancer were reported in either group. There was one case of lymphoma in the abatacept group and none in the placebo group. IR were computed and are presented in table 4.

In the cumulative double-blind and open-label abatacept exposure analysis, a total of 4134 patients with 8388 person-years of follow-up has been exposed to abatacept. IR of malignancies in the double-blind and cumulative periods were relatively similar; however, the rate of lung cancer was slightly lower in the cumulative study period. The age and sex-adjusted IR of total malignancies excluding NMSC in the abatacept clinical trials was 0.61 per 100 person-years (95% CI 0.45 to 0.80). This rate was similar to the age and sex-adjusted IR reported in the RA DMARD cohorts, except for the BC cohort, in which it was lower (table 5).

**Table 5:** Incidence of malignancies in the RA DMARD cohorts*

	BC	NDB	GPRD	NOAR	Sweden ERA
Total malignancies (excluding NMSC)	1274	NA^†^	472	30	148
	1.77 (1.51 to 2.08)		0.67 (0.51 to 0.87)	0.73 (0.56 to 0.93)	0.71 (0.55 to 0.92)
Breast cancer	205	156	94	4	13
	0.34 (0.24 to 0.49)	0.28 (0.19 to 0.42)	0.16 (0.09 to 0.28)	0.14 (0.08 to 0.25)	0.11 (0.06 to 0.21)
Colorectal cancer	139	52	40	2	20
	0.14 (0.08 to 0.24)	0.06 (0.03 to 0.15)	0.05 (0.02 to 0.13)	0.05 (0.02 to 0.13)	0.06 (0.02 to 0.14)^‡^
Lung cancer	218	116	109	4	23
	0.26 (0.17 to 0.39)	0.12 (0.07 to 0.22)	0.14 (0.08 to 0.25)	0.09 (0.04 to 0.18)	0.13 (0.07 to 0.23)
Lymphoma	94	35	41	3	11
	0.11 (0.06 to 0.21)	0.08 (0.04 to 0.17)	0.06 (0.02 to 0.14)	0.07 (0.03 to 0.16)	0.06 (0.02 to 0.14)

Values represent numbers of incident cancer cases and incidence rates (IR) per 100 person-years (95% CI). *Rates adjusted to the age and sex distribution of the abatacept clinical trial patients. ^†^This outcome was not available for the NDB. ^‡^The Sweden ERA cohort for the colorectal cancer analysis consists of 4295 patients rather than the 3703 used for the other malignancies.

BC, British Columbia RA Cohort; DMARD, disease-modifying antirheumatic drug; GPRD, General Practice Research Database; NDB, National Data Bank for Rheumatic Diseases; NMSC, non-melanoma skin cancer; NOAR, Norfolk Arthritis Register; RA, rheumatoid arthritis; Sweden ERA, Sweden Early Rheumatoid Arthritis Register.

Overall, except for the BC cohort in which higher IR were observed, there was considerable overlap of the 95% CI among the RA cohorts when compared with abatacept. The abatacept rate of breast cancer (0.08/100 person-years) was lower than the range of IR in the RA DMARD cohorts (0.11–0.34/100 person-years). The abatacept IR of lung cancer (0.15/100 person-years; 95% CI 0.08 to 0.27) and lymphoma (0.06/100 person-years; 95% CI 0.02 to 0.14) were within the range of IR from the five RA cohorts (0.09–0.26/100 person-years and 0.06–0.11/100 person-years for lung cancer and lymphoma, respectively).

**Figure 1:**
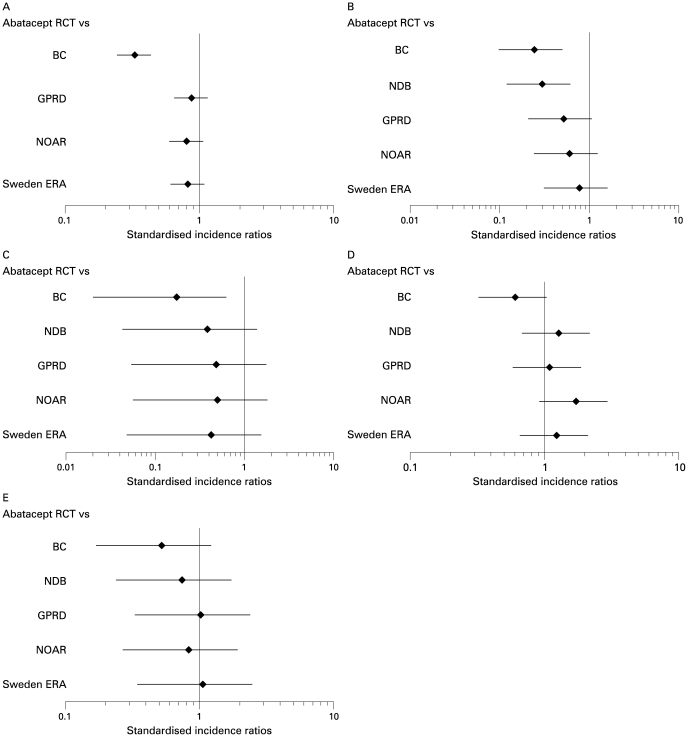
Standardised incidence ratios (SIR) of malignancies in the abatacept cumulative study periods compared with the observational rheumatoid arthritis disease-modifying antirheumatic drug cohorts. Values represent SIR and 95% CI. (A) Total malignancy (excluding non-melanoma skin cancer); (B) Breast cancer; (C) Colorectal cancer; (D) Lung cancer; (E) Lymphoma. BC, population-based British Columbia RA Cohort; GPRD, General Practice Research Database; NDB, National Data Bank for Rheumatic Diseases; NOAR, Norfolk Arthritis Register; RCT, randomised controlled trial; Sweden ERA, Sweden Early Rheumatoid Arthritis Register.

Figure 1 presents the SIR representing the relative risk of total malignancy (excluding NMSC) with abatacept compared with each of the RA cohorts. The SIR are somewhat reduced compared with the BC cohort, which had a higher incidence of cancers. The summary SIR comparing the rate of total malignancies (excluding NMSC) in the abatacept CDP with the pooled IR from the RA cohorts was 0.68 (95% CI 0.37 to 1.26), indicating that the overall risk of cancer was not significantly increased in abatacept-treated patients compared with RA patients treated with DMARD. Regarding specific types of cancers, the risk of breast cancer with abatacept may be reduced (summary SIR 0.42, 95% CI 0.18 to 1.00); however, women with RA were screened for prevalent breast cancer with mammography before enrollment in the double-blind abatacept studies. The summary SIR estimate for colorectal cancer was 0.33 (95% CI 0.08 to 1.44). The risk of lung cancer with abatacept was not significantly increased compared with any of the cohorts, although the point estimates were above 1 in the comparison with four of the five cohorts; the summary SIR estimate was 1.07 (95% CI 0.55 to 2.07). There appeared to be general risk parity for lymphoma between abatacept and the RA DMARD cohorts, although confidence intervals were wide. The overall summary SIR for lymphoma was 0.89 (95% CI 0.36 to 2.15).

**Figure 2:**
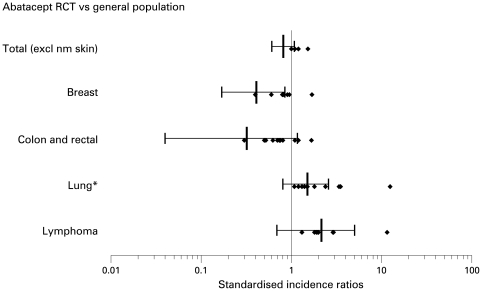
Standardised incidence ratios (SIR) of malignancies in the abatacept cumulative study periods compared with the general population (Surveillance Epidemiology and End Results (SEER)). The lines indicate the SIR point estimates and 95% CI for abatacept compared with the general population (SEER). The diamonds represent SIR reported in the literature that compare rheumatoid arthritis (RA) patients with non-RA patients or general populations. *One study had no observed cases of lung cancer; the SIR is not presented in the figure. RCT, randomised controlled trial.

For the comparison of the abatacept clinical trial malignancy experience with the general population, the calculated SIR comparing cancer IR in RA patients treated with abatacept with IR in the general population, from the SEER cancer registry, were 0.82 (95% CI 0.61 to 1.08) for total malignancy excluding NMSC, 0.41 (95% CI 0.17 to 0.85) for breast cancer, 0.32 (95% CI 0.04 to 1.16) for colorectal cancer, 1.51 (95% CI 0.80 to 2.59) for lung cancer and 2.17 (95% CI 0.70 to 5.07) for lymphoma. These SIR fell within the range of SIR published in studies comparing malignancy in RA samples with the general population (fig 2).^15^ ^21^ ^24^ ^25^ ^26^ ^27^ ^28^ ^29^ ^30^ ^31^ ^32^ ^33^ ^34^ ^35^ ^36^ ^37^ ^38^

## Discussion

This study provides context for the malignancy experience in patients treated with abatacept. The data presented suggest that the observed incidence of total malignancy (excluding NMSC) and of four site-specific malignancies (breast, colorectal, lung and lymphoma) in patients within the abatacept CDP is largely consistent with what may be expected based on cohorts of RA patients treated with non-biological DMARD. Similarly, the SIR estimates comparing the abatacept trial experience with that in the general population were similar to SIR comparing other RA cohorts with the general population.

The potential association between malignancies and RA has previously been reviewed.^8^ ^40^ Most studies suggest that the risk of total or solid malignancies in patients with RA is not substantially different compared with the general population or individuals without RA;^21^ ^22^ ^23^ ^24^ ^25^ ^26^ ^27^ ^28^ ^29^ ^30^ ^32^ ^36^ ^38^ however, data suggest that RA patients are at higher risks of lymphoma and lung cancer.^8^ Whereas the incidence of breast cancer may be slightly decreased in patients with RA,^24^ ^26^ ^29^ several studies found no significant difference when compared with the general population.^25^ ^27^ ^28^ ^30^ ^31^ A number of investigators have observed a decreased risk of colon or rectum cancer associated with RA, which may result from the long-term use of non-steroidal anti-inflammatory drugs (NSAID) or cyclooxygenase-2 selective inhibitors.^24^ ^25^ ^26^ ^27^ ^28^ ^29^ ^32^ Given these malignancy differences in RA patients, an RA cohort represents a more appropriate comparator for evaluating the malignancy risk of specific RA interventions than the general population.

A number of recent studies has examined malignancy risk in RA patients in the context of medication use. In their meta-analysis of nine clinical trials of infliximab or adalimumab, Bongartz *et al*^6^ suggested an increased risk of malignancy in the treatment arms compared with placebo. However, several large epidemiological studies found no increased risk in overall malignancy or solid malignancy associated with anti-tumour necrosis factor (TNF) use compared with non-use or methotrexate.^24^ ^26^ ^31^ ^38^ The majority of studies examining the effect of medication use on malignancy in RA have focused on lymphoma. Epidemiological studies have not documented a significantly elevated risk of lymphoma with anti-TNF use compared with non-use or methotrexate.^15^ ^31^ ^35^ ^38^ Several studies, however, have linked azathioprine use with lymphoma^41^ ^42^ and the data on methotrexate and lymphoma have been mixed.^43^ ^44^

This study has several strengths, including comparisons with a diversity of relevant RA populations from different sources as well as with the general population. A total of five large RA cohorts was identified and used to obtain a range of reference rates. When conducting pharmacoepidemiological studies, it has been recommended that at least two cohorts be evaluated to establish the extent of reproducibility.^45^ The resulting variation in the IR among the cohorts provides a more useful range of estimates for comparison than a single cohort IR. In our study, despite the geographical differences and the differences in ascertainment methods, the ranges of age and sex-adjusted IR were relatively narrow among the various RA cohorts, indicating good reproducibility of our results. Another strength of the study was the choice of comparison group. We were able to include in the study, as a reference group, only those patients treated with DMARD from the respective RA cohorts. Given that DMARD comprised the background therapy in all abatacept-treated patients in the trials, this constitutes an appropriate reference group for comparing the risk of malignancy.

This study has several potential limitations, including those inherent in the study design. The data collected and analysed from the RA cohorts were not primarily collected for this type of study. The limitations associated with the use of external control groups include but are not limited to differences in management and diagnoses of RA, the inclusion of both prevalent and new users of DMARD agents, differences in the ascertainment and verification of outcomes, length of follow-up, validity of RA diagnosis and severity of disease. We acknowledge that these variables may be different among the RA cohorts. Although the abatacept population appeared to be demographically similar to the cohorts, clinical trial patients are inherently different. The abatacept CDP enrolled mainly prevalent, stable non-biological DMARD users who have an inadequate response to their current therapy; thereby implying more severe disease in these patients. The RA cohort populations were a diverse group of both prevalent and new non-biological DMARD users. The RA cohort populations are potentially more stable in that this population had to be on a non-biological DMARD throughout follow-up without the addition of a biological therapy. However the non-biological DMARD treatment in these groups could be altered during follow-up such that non-biological DMARD therapy could be increased, decreased, or another non-biological treatment could be added to the current regimen all together. Trial patients may be monitored more closely for adverse events, which might overestimate our calculated SIR. However, some of the cohorts have prespecified observation times for patients enrolled in the registry (eg, NOAR). Malignancy screening for breast cancer with mammograms before entry in the trials may have resulted in a lower incidence of breast cancer in the abatacept CDP. The exclusion of patients with a history of cancer within the past 5 years of study entry suggests that enrolled patients are potentially healthier, resulting in a lower overall incidence of cancer. It is not uncommon for clinical trials to exclude patients who may be predisposed to cancer. There is also the concern of latency when evaluating malignancy outcomes. We were not able to adjust for potential confounders such as severity of disease, smoking, ethnicity, co-morbidities and the use of non-DMARD medications (ie, NSAID, corticosteroids) due to unavailability of data on these variables in most external cohorts. Finally, each of the databases used in this study may be associated with specific limitations, such as uncertainty surrounding diagnostic accuracy in administrative claims databases (eg, BC), small cohort size (eg, NOAR), the use of self-reporting (eg, NDB), etc. However, these individual limitations were minimised by the use of multiple cohorts, resulting in a range of references that nevertheless provided relatively consistent results.

In conclusion, external observational data provide useful information when long-term comparator data from randomised controlled trials are not available. Although differences in selection, surveillance and verification exist between clinical trials and observational cohorts, a range of estimates provides useful context for the evaluation of the abatacept long-term clinical trial experience. These data add to the continuing body of evidence evaluating the safety of abatacept and suggest that the observed number of malignancies is within the range of expected malignancies based on RA cohorts on a background of non-biological DMARD treatment. The overall safety of abatacept with respect to malignancies will continue to be monitored as part of a post-marketing surveillance programme.
